# BacT-Seq, a Nanopore-Based Whole-Genome Sequencing Workflow Prototype for Rapid and Accurate Pathogen Identification and Resistance Prediction from Positive Blood Cultures: A Feasibility Study

**DOI:** 10.3390/diagnostics16010133

**Published:** 2026-01-01

**Authors:** Meriem El Azami, Véronique Lanet, Corinne Beaulieu, Aurélien Griffon, Stéphane Schicklin, Pierre Mahé, Marion Darnaud, Marion Helsmoortel, Erwin Sentausa, Adrien Saliou, Mallory Poncelet, Raphaël Fleury, Marine Ibranosyan, François Vandenesch, Emmanuelle Santiago-Allexant

**Affiliations:** 1 BioMérieux SA, 69280 Marcy-l’Étoile, France; meriem.elazami@biomerieux.com (M.E.A.);; 2 BIOASTER, Microbiology Research Institute, 69007 Lyon, Francehelsmoortel@outlook.fr (M.H.); a.saliou@lesaffre.com (A.S.);; 3 University Hospital Microbiology Laboratory, Hospices Civils de Lyon, 69004 Lyon, France

**Keywords:** whole-genome sequencing, microbial identification, pathogen identification, antimicrobial susceptibility testing (AST), antimicrobial susceptibility prediction (ASP), antimicrobial resistance (AMR), blood culture, bloodstream infection (BSI), bacteremia, BacT-Seq pipeline

## Abstract

**Background/Objectives**: Rapid and accurate pathogen identification and antimicrobial susceptibility testing (AST) are critical for the proper management of patients with bloodstream infection (BSI). Real-time whole-genome sequencing (WGS) represents an attractive opportunity for exhaustive pathogen identification and antimicrobial susceptibility prediction (ASP). This feasibility study introduces BacT-Seq, a WGS-based prototype assay for the rapid and accurate identification of pathogens and the prediction of antimicrobial susceptibility from positive blood cultures using Oxford Nanopore Technologies (ONT) sequencing. **Methods**: A total of 200 positive blood culture samples from patients with a confirmed BSI were included in this study. DNA isolation from blood cultures was optimized prior to GridION (ONT) sequencing. Pathogen identification and several ASP methods were compared to conventional identification and phenotypic AST methods. **Results**: Most of the mono-microbial (89%) and poly-microbial (88%) samples were identified by BacT-Seq in less than 10 min of sequencing. While identification of poly-microbial samples remains challenging, identification of mono-microbial samples by sequencing was non-inferior to that of the conventional approach, even revealing an added value in terms of exhaustivity and/or taxonomic resolution. Machine-learning-based ASP models yielded 80% predictions in 2.5 h of sequencing. Their ability to predict resistance phenotypes varied with the microbial species evaluated, from 55/57 (96.5%) for *Escherichia coli* to 24/48 (50.0%) for *Pseudomonas aeruginosa*. **Conclusions**: This study demonstrates the feasibility of implementation of the BacT-Seq platform for the fast and accurate identification of pathogens from positive blood cultures. BacT-Seq performance of resistance predictions by bioinformatics tools is promising but requires further optimization and validation before clinical implementation.

## 1. Introduction

Bloodstream infections (BSIs) and sepsis are a major cause of morbidity and mortality worldwide, with sepsis-related deaths representing about 20% of all global deaths [1,2]. This healthcare burden is aggravated by the rising prevalence of antimicrobial resistance (AMR) [3,4], especially among infections by Gram-negative bacteria such as *Escherichia coli* (*E. coli*), *Klebsiella pneumoniae* (*K. pneumoniae*), and *Pseudomonas aeruginosa* (*P. aeruginosa*) or the Gram-positive bacteria *Staphylococcus aureus* (*S. aureus*) [5,6].

Rapid and accurate pathogen identification and antimicrobial susceptibility testing (AST) are critical to patient outcomes. They can reduce the time to initiate targeted therapy, the length of hospital stay, antibiotic use, and mortality, while reducing overall healthcare costs [7,8].

Conventional culture-based methods, still considered the gold standard for bacteremia diagnosis, take time (2–3 days or more to result) and are labor-intensive. Single-pathogen PCR and, more recently, multiplexing PCR syndromic panels allow the rapid and accurate detection of pathogens and AMR determinants from positive blood cultures, improving patient management compared to conventional methods [7,8,9,10,11,12,13,14,15,16,17,18,19,20].

Despite significant improvements, multiplexing PCR presents some limitations, the main one being that it only detects targets included on the panel. This increases the risk of missing the detection of clinically relevant pathogens or of AMR markers, while requiring a recurrent update to the syndromic panels to follow the evolution of AMR determinants. These limitations highlight the need for more comprehensive approaches, such as real-time whole-genome sequencing (WGS).

Real-time whole-genome sequencing (WGS) represents an attractive opportunity for exhaustive pathogen identification and antimicrobial susceptibility prediction (ASP), resulting in a more accurate diagnosis and thus potentially improving patient management. A key advantage of WGS, especially over conventional phenotypic AST—which is performed on a single-colony isolate and therefore overlooks population heterogeneity—is that it can interrogate the entire microbial population within a clinical sample. This enables the detection of resistance determinants in low-frequency subpopulations that may drive treatment failure.

A few studies recently evaluated the feasibility of WGS-based bacteremia diagnosis from positive blood cultures [21,22,23,24,25]. These studies demonstrated the superiority of nanopore sequencing technology (Oxford Nanopore Technologies [ONT]) [22,23,24,25] over Illumina [21], notably in terms of time to results and the benefit of depleting host genomic DNA prior to DNA extraction to improve the quality of sequenced microbial DNA [21,22,25]. While most existing feasibility studies used samples spiked with selected bacterial isolates (including *E. coli*, *S. aureus*, *K. pneumoniae*) [22,23,24], only a few used clinical positive blood culture samples [21,25]. Taxonomic identification of sequencing data is usually achieved against existing public databases (such as BLAST, kraken2, RefSeq) [21,22,23,24,25], and ASP is performed either by direct association using open-source databases (such as ResFinder, CARD, ABRicate) [22,23,24] or using machine-learning algorithms (AREScloud) [21,25].

Major challenges in achieving the routine implementation of WGS for pathogen identification and ASP testing include technical optimization of sequencing and improvement of bioinformatic analysis of sequencing data, to obtain accurate and easy-to-interpret results in a short period of time.

The present feasibility study describes BacT-Seq, a WGS-based prototype assay for the rapid and accurate identification of pathogens and the prediction of antimicrobial susceptibility from clinical positive blood cultures. We present the results of assay optimization, the performance of BacT-Seq analyses compared to those of a conventional reference approach, and the resulting time to result (TTR) for pathogen identification and machine learning-based ASP.

## 2. Materials and Methods

### 2.1. Study Samples and Design

Positive blood cultures from patients with a confirmed bloodstream infection were included in this study. Other inclusion criteria were the availability of microbial identification results obtained using VITEK^®^ MS (bioMérieux, Marcy-l’Étoile, France) and the availability of antimicrobial susceptibility testing (AST) results based on VITEK^®^ 2 (bioMérieux) and/or another validated reference method. Exclusion criteria included patient age < 18 years and pregnancy. Two sets (the pre-optimization set and optimization set) of 200 positive blood culture samples (of at least 21 mL each) were provided by a reference microbiology laboratory (University Hospital Microbiology Laboratory, Hospices Civils de Lyon [HCL], Lyon, France). Samples were selected to cover the most prevalent microbial species, as well as rare species, while reducing the natural over-representation of some species (e.g., coagulase-negative staphylococci). Hence, samples selected for this study aimed to cover a broad range of relevant species and did not reflect the true local pathogen epidemiology. Samples were anonymized and stored at 4 °C ≤48 h before being shipped to bioMérieux (Marcy-l’Étoile, France) for further processing. DNA was extracted at bioMérieux then shipped in batches to BIOASTER Technology Research Institute (Lyon, France) for sequencing. Bioinformatics analysis of sequencing data was performed at bioMérieux using the prototype BacT-Seq pipeline.

Positive blood culture samples were leftover specimens from individual patients previously tested by the reference lab for the diagnosis of BSI. Consequently, no ethics approval was required for this study. In accordance with French legislation, a CODECOH declaration (describing the activity of preparation and conservation of leftover samples for research purposes) was deposited at the French Ministry of Higher Education and Research, and all patients were informed of the possible use of leftover samples for research purposes and expressed their non-opposition to their use.

### 2.2. Blood Culture

Microbial blood cultures were conducted by the reference microbiology lab (HCL) using the automated BACT/ALERT^®^VIRTUO^®^ system (bioMérieux, Marcy-l’Étoile, France), according to the recommended protocol. The time to positivity for bacterial cultures using BACT/ALERT^®^VIRTUO^®^ is about 12–24 h [26]. In this study, 159/188 (84.6%) mono-microbial samples were positive within 24 h, and 75/188 (39.9%) within 12 h, thus in range with the expected time to positivity. In this study, only one positive blood culture was obtained per patient (i.e., each of the 200 positive blood cultures included in the analysis originated from a different patient). This also means that poly-microbial specimens described in this study came from the same culture bottle.

### 2.3. Reference Pipeline for Phenotypic Microbial Identification and Antimicrobial Susceptibility Testing

Conventional identification and phenotypic analyses were conducted by the reference lab (HCL) prior to the start of the study. These data served as a reference for comparison with sequencing-based genotypic analyses.

#### 2.3.1. Microbial Identification by Reference Method

Microbial identification was performed on overnight subcultures by MALDI-TOF using the VITEK^®^ MS system (bioMérieux), according to the manufacturer’s instructions.

#### 2.3.2. Phenotypic Antimicrobial Susceptibility Testing

AST was applied to isolated colonies obtained after subcultures of positive blood cultures using the following validated reference methods: (i) VITEK^®^ 2 (bioMérieux) for most species, (ii) disk-diffusion, (in-house assay using SirScan Discs, i2a S.A., Intelligence Artificielle Applications, Montpellier, France), and (iii) ETEST^®^ (bioMérieux) for certain species (e.g., *Streptococcus* spp.). Based on the minimum inhibitory concentration (MIC) of antibiotics provided by the AST method, three phenotypes were issued according to the 2019 breakpoint guidelines of the Antibiogram Committee of the French Society for Microbiology (CA-SFM) in collaboration with the European Committee on Antimicrobial Susceptibility Testing (EUCAST) (CA-SFM/EUCAST 2019): resistant (R), susceptible (S), and intermediate (also known as ‘susceptible, increased exposure’) (I). I phenotypes were excluded from the comparative analysis with R/S phenotypes predicted by the BacT-Seq sequencing pipeline.

### 2.4. BacT-Seq Sequencing Pipeline for Genotypic Microbial Identification and Antimicrobial Resistance Detection and/or Prediction

#### 2.4.1. BacT-Seq Pipeline Description

**Figure 1:**
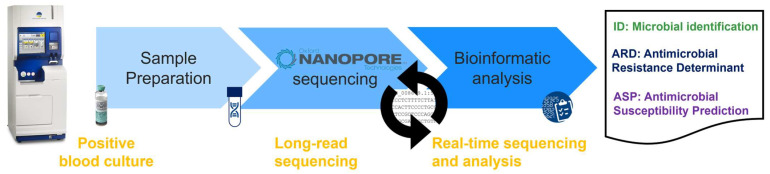
BacT-Seq sequencing platform workflow. Sample preparation from a positive blood culture includes microbial DNA enrichment followed by DNA extraction and library preparation for nanopore sequencing. Bioinformatic analysis of sequencing data yields microbial identification and resistance prediction (via the antimicrobial resistance determinant [ARD] detection and the antimicrobial susceptibility prediction [ASP] approaches).

The current prototype version of the BacT-Seq platform integrates sample preparation optimization followed by real-time sequencing using nanopore technology (see Section 2.4.3) and bioinformatic analysis for genotypic identification and resistance prediction based on two approaches: (i) resistance detection by direct comparison with resistance markers described in the literature (antimicrobial resistance determinant [ARD] detection) and (ii) resistance prediction based on machine-learning models (antimicrobial susceptibility prediction [ASP]) (Figure 1).

The bioinformatics pipeline includes seven components: (i) an Input Data Handling component to check the integrity of the input fastq files and format them for further analysis, (ii) a QC and Filtering component, (iii) a Taxonomic Binning component, based on kraken2 and a custom reference database, to assign reads to microbial (bacteria, fungi, viruses) or human taxonomy, (iv) a Plasmid Identification component to identify plasmid reads and improve the detection of microbial resistance, (v) a Microbial Detection component to identify microorganisms (see Section 2.4.4), (vi) an ARD component to detect antimicrobial resistance determinants (see Section 2.4.5), and (vii) an ASP component to predict antimicrobial susceptibility and resistance (see Section 2.4.6).

#### 2.4.2. Microbial DNA Enrichment

Microbial DNA enrichment from positive blood cultures was carried out using a prototype version of the VITEK^®^ MITUBE™ device (bioMérieux, Marcy-l’Étoile, France). VITEK^®^ MITUBE™ (thereafter referred to as “MITUBE”) is a disposable sample preparation device that concentrates microorganisms present in a positive blood culture sample into a pellet, while partially eliminating human cells in the process. This concentration of microorganisms by MITUBE enables their rapid identification directly from positive blood culture bottles, such as those from BACT/ALERT^®^ [27].

In the context of BacT-Seq, the resulting pellet serves as input sample for the subsequent DNA extraction step (as described in Section 2.4.3). In this research project (as presented in Section 3.3, Section 3.4, Section 3.5 and Section 3.6), the MITUBE prototype was implemented to reduce the amount of human DNA from the blood culture, thereby increasing the proportion of microbial DNA available for sequencing.

#### 2.4.3. Sample Preparation and Sequencing

Prior to sequencing, DNA was extracted using the ZymoBIOMICS DNA Miniprep kit (Zymo Research, Freiburg, Germany) either directly from positive blood cultures (pre-optimization batch) or from positive blood cultures processed with the MITUBE system (see Section 2.4.2; optimization batch). Sequencing was performed at BIOASTER Technology Research Institute (Lyon, France) as follows. Libraries were prepared using the Rapid Barcoding Sequencing kit (ref. SQK-RBK004; Oxford Nanopore Technologies [ONT], Oxford, UK). Pools of four samples were loaded on a SpotOn Flow Cell Rev D R9.4.1 (ref. FLO-MIN106D; ONT) with more than 1100 active pores at quality check. A GridION X5 sequencer (ONT) was set for 72 h runs monitored by the latest MinKNOW core version at the time (v4.2.5 to 5.2.4) with high accuracy basecalling (HAC) using Guppy (v4.3.4 to 6.2.11). The first 150 and last 50 nucleotides of each read were trimmed, and reads with an average quality score below 7 were filtered using NanoFilt v2.7.1 [28]. Lambda phage reads were removed with NanoLyse v1.2.0 [28].

#### 2.4.4. Microbial Identification by BacT-Seq

Microbial identification was performed by the Microbial Detection component of BacT-Seq, which is based on the results of the Taxonomic Binning component of the pipeline. Time to identification was calculated as the sequencing time (in minutes) necessary to obtain an identification by the BacT-Seq pipeline.

#### 2.4.5. Antimicrobial Resistance Determinant Detection by Direct Association

The ARD component of the BacT-Seq pipeline detects antimicrobial resistance determinants in two steps. First, a genotypic analysis of the sequencing reads is performed. Minimap2 is used to align the reads using the option “-x map-ont”, which conditions the use of parameters optimized for long, noisy reads from ONT sequencing [29]. The candidate markers are then filtered by considering a minimum depth of coverage of 5x and a minimum breadth of coverage of 99%. This step searches for candidate ARDs (i.e., marker sequences and point mutations) against a custom ARD database built and updated from public databases embedded in open-source search tools, including AMRFinderPlus [30], ResFinder [31], and PointFinder [32]. In a second step, a “direct association” strategy is adopted to infer resistance, considering all ARDs that were not filtered in the first step. Thus, the presence of any of these ARDs in a given sample leads to the prediction of the resistance (R) phenotype for the impacted antibiotics.

For this study, 3346 R/S phenotypes were compared across 24 microbial species. Two levels of analyses were conducted: according to drug-level annotations (the ARD impacts a given antibiotics) and according to family-level annotations (the ARD impacts at least one member of an antibiotic family).

#### 2.4.6. Antimicrobial Susceptibility Prediction Models

As opposed to the ARD component, which detects antimicrobial resistance or susceptibility (R/S) by comparison with known resistance markers, the ASP component of the BacT-Seq pipeline predicts R/S phenotypes based on a machine-learning approach. Machine-learning models were trained on datasets composed of genomes (assemblies) and associated resistance phenotypes for selected drugs and species. Resistance phenotypes used to build ASP models are based on the 2019 Clinical and Laboratory Standards Institute (CLSI) clinical breakpoints. For this study, we were able to build training datasets for four species (*S. aureus*, *E. coli*, *K. pneumoniae* and *P. aeruginosa*) and 7 to 11 antibiotics (Table S1). These four bacterial species are most frequently found in positive blood culture samples and are also well represented in public databases such as NCBI and PATRIC [33,34,35]. Four machine-learning models were built, including lasso-penalized logistic regression [36], cluster-lasso [37], stability [38], and XGBoost [39]. To train these machine-learning models, each genome was represented by a vector of binary variables encoding the presence or the absence of every possible k-mer (sequence of length k) in the genome. The k-mer representation was computed using the DBGWAS software [40]. A 10-fold cross-validation strategy was used to optimize model parameters and to select the best model among the four machine-learning models. For this study, all samples with an identified species within the ASP panel (i.e., *S. aureus*, *E. coli*, *K. pneumoniae*, or *P. aeruginosa*) underwent ASP analysis. For a given sample, the best machine-learning model was used to predict resistance (R) or susceptibility (S) to all available drugs.

### 2.5. Data Analysis

#### 2.5.1. Performance Evaluation Metrics

Microorganism identification by the BacT-Seq pipeline was compared to that by the reference identification platform using a confusion matrix. Discrepancies were verified and confirmed by the reference lab. Accuracy was defined as the percentage of samples with a sequencing-based identity in perfect concordance with the reference method (exact same identification). Sensitivity was defined as the percentage of samples with a sequencing result concordant with that of the reference method at the species level (reference species identification).

ARD and ASP results for resistance (R) and susceptibility (S) phenotypes were compared to those obtained with the reference AST platform using a confusion matrix. Intermediate (I) phenotypes identified by the reference AST method were excluded from the ARD/ASP performance analyses. Specificity was defined as the proportion (%) of S reference phenotypes predicted as S by BacT-Seq. Sensitivity was defined as the proportion (%) of R reference phenotypes predicted as R by BacT-Seq. Reference R phenotypes predicted as S were defined as very major errors (VMEs), while reference S phenotypes predicted as R were defined as major errors (MEs).

#### 2.5.2. Time-to-Result (TTR) Analyses

TTR for pathogen identification and ASP was defined as the sequencing time necessary to obtain an identification and an ASP result by the BacT-Seq sequencing platform, respectively. Time to ASP was analyzed by two different approaches, using either 50X sequencing depth coverage sequencing data (identified as sufficient to reach a plateau of performance in preliminary investigations) or an adaptive procedure to reduce the amount of sequencing data required for an accurate ASP (as an optimization approach to reduce time to result). Time to ASP results were displayed in Tukey box plots generated using GraphPad Prism 5.04 (GraphPad Software, San Diego, CA, USA), together with the respective median and interquartile range (IQR) values. TTR for ASP was also evaluated using cumulative frequency.

## 3. Results

### 3.1. Characteristics of Microbial Samples According to Reference Methods

#### 3.1.1. Reference Identification of Microbial Samples

Microbial identification in the 200 positive blood culture samples was performed on subcultured colonies using VITEK MS as the reference method. Of the 200 samples, 188 (94.0%) were identified as mono-microbial and 12 (6.0%) as poly-microbial. Of the 12 poly-microbial samples, 10 were bi-detections and 2 were tri-detections (Table 1).

**Figure 2:**
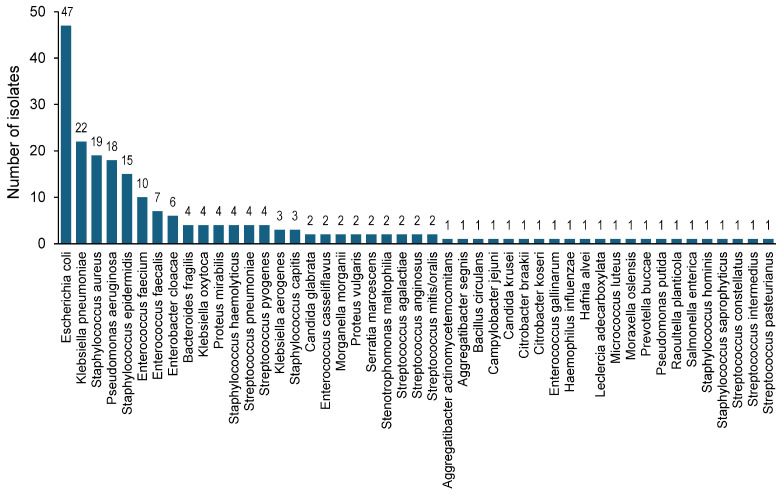
Distribution per species of the 214 microbial isolates identified by the VITEK MS reference method (n = 47 species). The four most represented bacterial species are *E. coli* (47 isolates), *K. pneumoniae* (22 isolates), *S. aureus* (19 isolates), and *P. aeruginosa* (18 isolates). It should be noted that this distribution does not reflect the true pathogen epidemiology, due to sampling bias (see Section 2.1).

A total of 214 isolates from 26 genera and 47 species were identified (Figure 2). Most (211/214 [98.6%]) were bacterial isolates, and 3/214 (1.4%) were fungal isolates (Candida genus). Almost half (106/214 [49.5%]) of the isolates were represented by the four bacterial species *E. coli* (47 isolates), *K. pneumoniae* (22 isolates), *S. aureus* (19 isolates), and *P. aeruginosa* (18 isolates) (Figure 2).

#### 3.1.2. Reference Antimicrobial Susceptibility Testing

**Figure 3:**
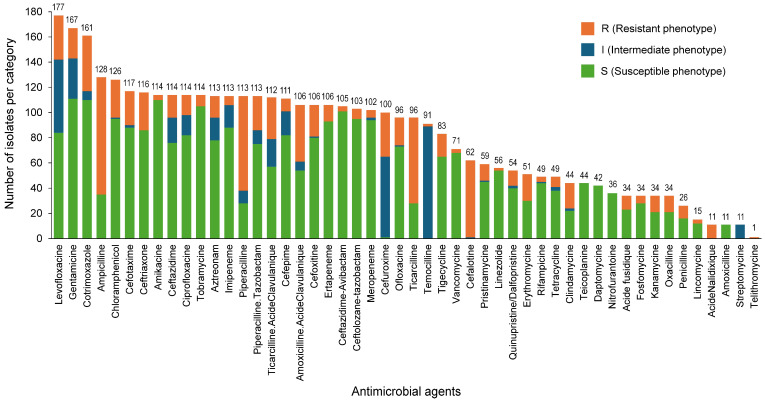
Antimicrobial susceptibility testing (AST). Distribution of the resistant (R), susceptible (S), and intermediate (I) phenotypes of the 185 isolates characterized by the reference AST methods (VITEK 2, disk-diffusion, and ETEST), across 50 evaluated antibiotics. Of note, reference AST was performed on single colonies obtained after subcultures of positive blood cultures (hence not assessing population heterogeneity).

Phenotypic AST was applied to the 214 identified isolates using validated reference methods. Of the 214 isolates, 185 (86.4%) were attributed an AST phenotype (170 using VITEK 2 and 15 using disk-diffusion and ETEST) and 29/214 (13.6%) isolates had no AST result. The distribution of the resistance, intermediate, and susceptibility (R/I/S) phenotypes for the 185 characterized isolates over 50 tested antibiotics is shown in Figure 3.

### 3.2. Genotypic Characterization of Microbial Samples by the BacT-Seq Sequencing Platform: Sample Preparation

**Figure 4:**
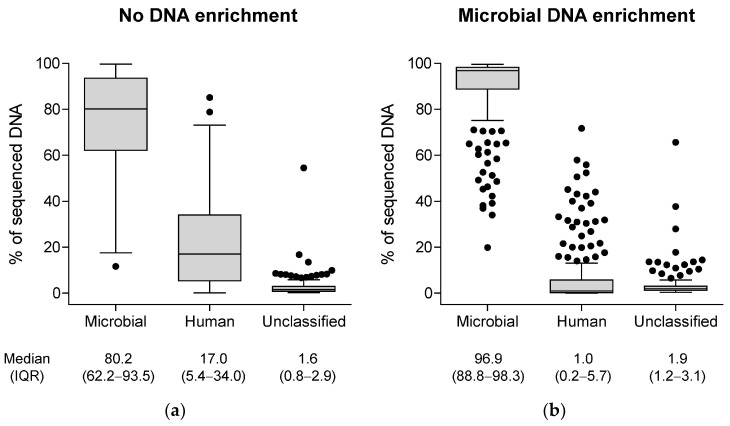
Distribution of the fraction of sequenced DNA that was assigned to the microbial super-kingdom or the human super-kingdom or was left unclassified by the Taxonomic Binning component of the BacT-Seq pipeline. (**a**) Samples not processed with MITUBE prior to DNA extraction and sequencing (n = 200) vs. (**b**) MITUBE-processed samples prior to DNA extraction and sequencing (n = 200). Tukey box plots with median and interquartile range (IQR) indicated below each plot.

The presence of contaminating DNA is a major source of data misinterpretation in next-generation sequencing, especially in studies aiming to identify genetic variants [41]. To optimize the quality of the sequencing data, we sought to enrich samples in microbial DNA by depleting contaminating human DNA prior to DNA extraction and sequencing. A set of 200 positive blood culture samples was processed through a MITUBE concentrating device (see Section 2.4.2) prior to DNA extraction. The resulting sequencing data were compared to those obtained from 200 independent positive blood culture samples directly subjected to DNA extraction. The taxonomic constitution of sequenced DNA from the two sets of samples was compared using the Taxonomic Binning component of the BacT-Seq pipeline (Figure 4a vs. Figure 4b).

Implementation of the MITUBE device on positive blood culture samples resulted in the enrichment of sequenced microbial DNA from a median (IQR) proportion of 80.2% (62.2–93.5) to 96.9% (88.8–98.3) and the depletion of sequenced human DNA from a median (IQR) proportion of 17.0% (5.4–34.0) to 1.0% (0.2–5.7) (Figure 4).

Because of its ability to enrich microbial DNA from positive blood culture samples, the MITUBE processing step is now part of the current version of the BacT-Seq procedure. All performance analyses described thereafter were conducted on samples processed with the MITUBE prototype device.

### 3.3. Performance of BacT-Seq-Based Microbial Identification vs. Reference Identification

#### 3.3.1. Mono-Microbial Samples

**Figure 5:**
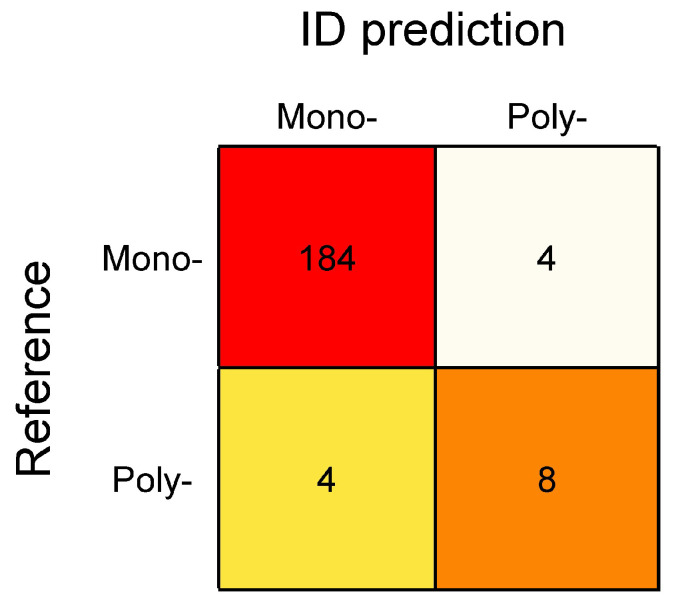
Confusion matrix of mono- and poly-microbial identifications predicted by BacT-Seq vs. the reference method.

Of the 188 samples identified as mono-microbial by the reference method, 184 (97.9%) were also identified as mono-microbial by BacT-Seq and 4 (2.1%) as poly-microbial (Figure 5). Of the latter, three involved the correct species together with an additional one, and one sample was assigned to the species group together with another species (Table 2).

Of the 184 mono-microbial samples identified as such by BacT-Seq, 168 (91.3%) were perfect matches (i.e., they provided the exact same identification as VITEK MS), 10 (5.4%) provided a more accurate identification by sequencing than the reference method (Table 3), and 6 (3.3%) showed the following discrepancies. Four of the six discrepancies corresponded to samples identified by sequencing either at the species group rank (*Streptococcus anginosus* group) or the genus rank (*Streptococcus*, *Bacillus*, *Prevotella*), and two samples were mis-identified by sequencing (*Aggregatibacter* aphrophilus vs. Aggregatibacter *segnis* by the reference method, and *Serratia* ureilytica vs. Serratia *marcescens* by the reference method) (Table 4).

Assay accuracy (defined as the fraction of samples in perfect concordance) was estimated at 94.7% ([168 + 10]/188). Assay sensitivity (defined as the fraction of samples with a correct species identification) was estimated at 96.3% ([168 + 10 + 3]/188).

#### 3.3.2. Poly-Microbial Samples

Of the 12 samples identified as poly-microbial by the reference method, 8 (66.7%) were also designated as polymicrobial by BacT-Seq, and 4 (33.3%) as mono-microbial (Figure 5). These four sequenced samples correctly identified one of the two species (Table 5; samples numbered 9 to 12) and missed the second one.

Of the eight poly-microbial samples identified as such by BacT-Seq, five (62.5%) were perfect matches (i.e., exact same identification of both species as with VITEK MS) (Table 5; samples numbered 1 to 5), and three (37.5%) showed the following discrepancies. In one sample with three detections by the reference method, two of them were identified by sequencing (the third one being missed by BacT-Seq) (Table 5; sample number 6). In another sample with three detections by the reference method, two bacterial strains were detected by sequencing, one of which was concordant with the reference method (thus one missed and one discordant) (Table 5; sample number 7). Finally, in one sample with two detections by the reference method, three detections were identified by sequencing, of which two were concordant (*Gemella morbillorum* not detected by VITEK MS) (Table 5; sample number 8).

#### 3.3.3. Time to Microbial Identification by BacT-Seq

**Figure 6:**
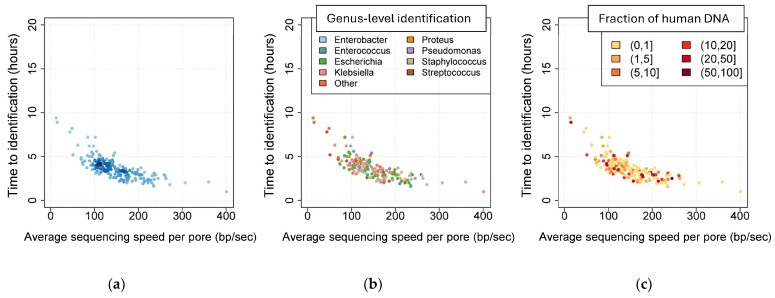
Time to microbial identification. Time to identification (in minutes) using the BacT-Seq pipeline for mono-microbial identifications, relative to the sequencing speed (per pore in bp/second) during the first hour of sequencing. Each point corresponds to a sample. (**a**) Blue color intensity reflects point density. (**b**) Color code according to the bacterial genus. (**c**) Color code according to the percentage of human DNA detected in the sample.

Time to microbial identification was calculated on 188 samples with mono-detections (184 true + 4 false mono-microbial samples; Figure 5). Time to identification was defined as the sequencing time (in minutes) necessary to obtain an identification by the BacT-Seq platform. All samples but one (187/188 [99.5%]) were identified in <10 min and 167/188 (88.8%) in ≤5 min (Figure 6). One *Campylobacter* sample was identified in 47 min of sequencing.

The time to microbial identification correlated with the sequencing speed (defined as the average sequencing speed per pore during the first hour of sequencing; Figure 6a) but was not associated with the bacterial species identified (Figure 6b) nor the fraction of human DNA sequenced (Figure 6c).

Comparable results were obtained for the 12 poly-microbial samples identified by BacT-Seq (8 true + 4 false poly-microbial samples; Figure 5, Table 2 and Table 5 [sample number 1 to 8]). Of the 25 detected bacterial species, 22 (88.0%) were detected in <10 min, 2 (8.0%) in <15 min, and 1 (4.0%; *Klebsiella oxytoca*) in 32 min.

### 3.4. Performance of BacT-Seq-Based Antimicrobial Resistance Determinant Detection vs. Reference Antimicrobial Susceptibility Testing

**Figure 7:**
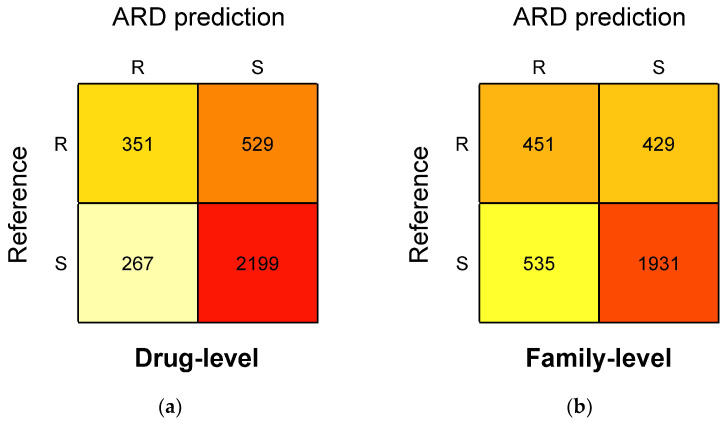
Confusion matrix of direct-association-based antimicrobial resistant determinant (ARD) predictions applied to all BacT-Seq data vs. antimicrobial susceptibility testing (AST) reference results. ARD detection results were generated according to drug-level annotations (impact on a given antibiotics) (**a**) and to family-level annotations (impact on at least one member of an antibiotic family) (**b**).

The direct-association-based ARD approach considers a priori knowledge of resistance markers (consolidated into a custom database) and is thus expected to cover a large number of microbial species, resistance markers, and antimicrobial agents. A total of 3346 R/S phenotypes (880 R and 2466 S) were predicted across 24 species using the ARD approach. Two ARD prediction analyses were conducted, considering either drug-level or antibiotic family-level associations. These ARD predictions were compared to the AST phenotypes obtained by the reference method (Figure 7).

Considering drug-level associations, 2550/3346 (76.2%) phenotypes were concordant, including 2199/2466 (89.2%) concordant S phenotypes and 351/880 (39.9%) concordant R phenotypes (Figure 7a). Of the 796 discordant predictions, 529 were reference R phenotypes predicted as S by ARD (also defined as very major errors [VMEs]) and 267 were reference S phenotypes predicted as R by ARD (defined as major errors [MEs]).

Considering less precise associations (at antibiotic family level), 2382/3346 (71.2%) phenotypes were concordant, including 1931/2466 (78.3%) concordant S phenotypes and 451/880 (51.2%) concordant R phenotypes (Figure 7b). Of the 964 discordant predictions, 429 were VMEs and 535 were MEs.

Altogether, the ARD approach was slightly better at predicting S phenotypes (specificity of 78.3% to 89.2%) than R phenotypes (sensitivity of 39.9% to 51.2%) (Table 6). Of the two levels of association, the less precise family-level associations were slightly better in predicting R phenotypes (sensitivity of 51.2%) but remained poorly predictive.

The “direct association” strategy of the ARD approach makes it difficult to finely control the trade-off in terms of the sensitivity and specificity of the model. Indeed, while increasing the number of resistance markers considered improves sensitivity, this is automatically at the detriment of specificity (by falsely predicting some S phenotypes as R). For this reason, an alternative ASP approach relying on machine learning and operating from a k-mer representation of the genomes was also tested.

### 3.5. Performance of BacT-Seq-Based Antimicrobial Susceptibility Prediction Models vs. Reference Antimicrobial Susceptibility Testing

**Figure 8:**
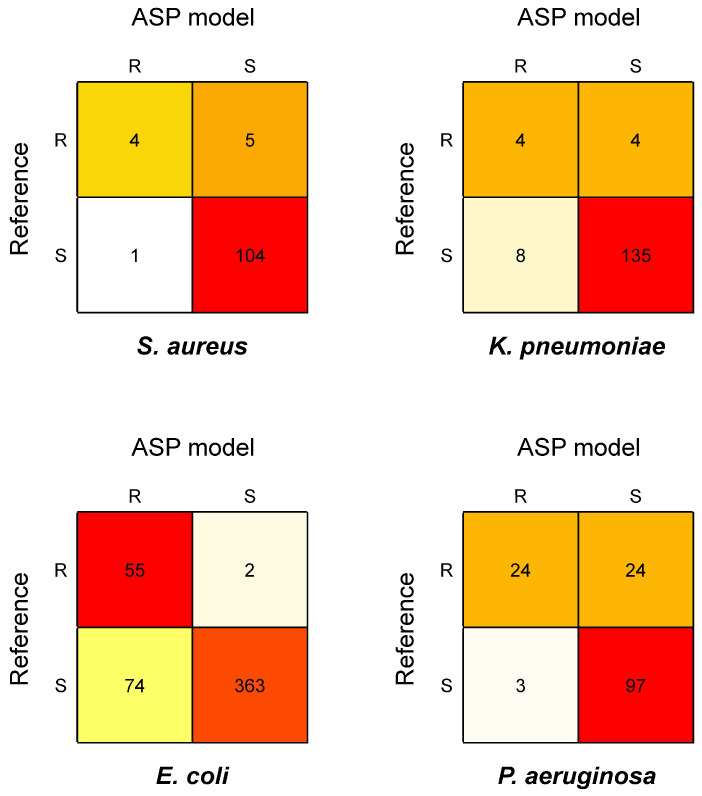
Confusion matrix of machine-learning-based antimicrobial susceptibility prediction (ASP) models applied to selected BacT-Seq data vs. antimicrobial susceptibility testing (AST) reference results. Four ASP models were developed for each of the following bacterial species: *S. aureus*, *K. pneumoniae*, *E. coli*, and *P. aeruginosa*. The results of the best predictive model are presented as a confusion matrix.

This exploratory approach is based on the construction of predictive models using machine learning. Four different models were trained on genomic and resistance phenotype data from the most frequent bacterial species (*E. coli*, *K. pneumoniae*, *P. aeruginosa*, and *S. aureus*), to generate ASP signatures specific to species/drug combinations. The best-performing model based on cross-validation results was chosen for the analysis (see Section 2.4.6). ASP results of selected samples (for which the respective bacterial species were detected by BacT-Seq) were compared to the respective AST reference phenotypes (Figure 8).

#### 3.5.1. ASP Signature for *S. aureus*

For the ASP analysis of *S. aureus*, 19 samples and 7 antibiotics (erythromycin, gentamicin, oxacillin, levofloxacin, tetracycline, oxacillin, and vancomycin) were considered. Of the 133 analyzed phenotypes, 19 with the I reference phenotype were excluded from the comparison. Of the 114 R/S phenotypes included in the comparison, 108 (94.7%) were concordant, including 104/105 (99.0%) concordant S phenotypes and 4/9 (44.4%) concordant R phenotypes (Figure 8, *S. aureus*). Of the six discordant predictions, five were defined as VME and one as ME. This single ME involved predicting as resistant an *S. aureus* strain defined as susceptible to clindamycin by the AST reference method. Of the five VMEs, three involved predicting as susceptible an *S. aureus* strain resistant to erythromycin, one a strain resistant to clindamycin, and one a strain resistant to tetracycline.

#### 3.5.2. ASP Signature for *K. pneumoniae*

For the ASP analysis of *K. pneumoniae*, 22 samples and eight antibiotics (amikacin, aztreonam, cefepime, cefoxitine, ceftazidime, ciprofloxacin, imipenem, meropenem) were considered. Of the 155 analyzed phenotypes, 4 with the I reference phenotype were excluded from the analysis. Of the 151 R/S phenotypes included in the comparison, 139 (92.0%) were concordant, including 135/143 (94.4%) concordant S phenotypes and 4/8 (50.0%) concordant R phenotypes (Figure 8, *K. pneumoniae*). Of the 12 discordant predictions, 4 were VMEs and 8 were MEs. Of the eight MEs, six involved predicting as resistant a *K. pneumoniae* strain susceptible to cefoxitine.

#### 3.5.3. ASP Signature for *E. coli*

For the ASP analysis of *E. coli*, 47 samples and 11 drugs (amoxicillin/clavulanic acid, ampicillin, cefotaxime, ceftazidime, ceftriaxone, cefuroxime, chloramphenicol, ciprofloxacin, levofloxacin, meropenem, and tobramycin) were considered. Of the 512 phenotypes, 18 with the I reference phenotype were excluded. Of the 494 R/S phenotypes considered in the comparison, 418 (84.6%) were concordant, including 363/437 (83.1%) S phenotypes and 55/57 (96.5%) R phenotypes (Figure 8, *E. coli*). Of the 76 discordant predictions, 2 were VMEs and 74 were MEs. The two VMEs involved predicting as susceptible one *E. coli* strain resistant to ampicillin and the other resistant to chloramphenicol. Most (62/74 [83.8%]) MEs involved four antibiotics, namely amoxicillin/clavulanic acid (17/62), cefotaxime (13/62), ceftazidime (16/62), and cefuroxime (16/62).

#### 3.5.4. ASP Signature for *P. aeruginosa*

For the ASP analysis of *P. aeruginosa*, 18 samples and 11 drugs (amikacin, aztreonam, cefepime, ceftazidime, ciprofloxacin, imipenem, levofloxacin, meropenem, piperacillin/tazobactam, ticarcillin/clavulanic acid, and tobramycin) were considered. Of the 166 phenotypes, 18 with the I reference phenotype were excluded. Of the 148 R/S phenotypes included in the comparison, 121 (81.8%) were concordant, including 97/100 (97.0%) S phenotypes and 24/48 (50.0%) R phenotypes (Figure 8, *P. aeruginosa*). Of the 27 discordant predictions, 24 were VMEs and 3 were MEs. The 24 VMEs involved all evaluated antibiotics, indicating a poorer performance of the ASP approach for *P. aeruginosa*. 

Altogether, ASP models performed better in predicting S than R phenotypes (higher specificity than sensitivity), except for the ASP model developed for *E. coli*, which showed both high sensitivity (96.5%) and specificity (83.1%) (Table 7).

With 24/48 (50.0%) sensitivity, the ASP model for *P. aeruginosa* was the least performant in predicting R phenotypes (not considering sensitivity calculations for *S. aureus* and *K. pneumoniae*, which are based on low case numbers).

### 3.6. Time to ASP Using the BacT-Seq Pipeline

**Figure 9:**
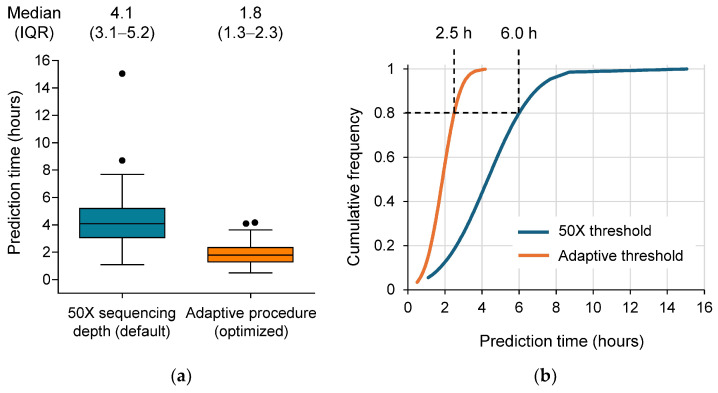
Time to antimicrobial susceptibility prediction (ASP) result, defined as the sequencing time (in hours) necessary to obtain a prediction. (**a**) Box plot of the distribution of the time to ASP of R/S phenotypes using the BacT-Seq pipeline (50X sequencing depth threshold vs. adaptive threshold procedure), considering the four species-specific ASP models (n = 104). Tukey box plots with median and interquartile range (IQR) indicated above each plot. (**b**) Cumulative frequency of the prediction times shown in panel (**a**).

The time to prediction by the machine-learning ASP approach of the BacT-Seq pipeline was calculated considering all samples for which a prediction was obtained for the four selected species (*S. aureus*, *K. pneumoniae*, *E. coli*, and *P. aeruginosa*) and for which a reference AST prediction was available (n = 104). The time to ASP was defined as the sequencing time (in hours) necessary to obtain a prediction. To optimize the time to ASP by BacT-Seq, two procedures were implemented in the ASP component of the bioinformatics pipeline and compared. The first procedure considered sequencing results achieving 50X sequencing depth (i.e., high sequencing coverage). An adaptive procedure was also adopted to reduce the amount of sequencing data (i.e., reduced sequencing depth) required to make an accurate ASP (Figure 9).

The time to ASP result for the 104 included samples ranged from 1.1 to 15.0 h using the 50X threshold procedure, with a median (IQR) time of 4.1 (3.1–5.2) hours. In comparison, the time to ASP using the adaptive procedure ranged from 0.5 to 4.2 h, with a median (IQR) time of 1.8 (1.3–2.3) hours (Figure 9a). Considering cumulative frequency, 80% of the ASP results were available in 6.0 h with the 50X threshold procedure vs. 2.5 h using the adaptive procedure (Figure 9b).

Given that pre-sequencing steps require about 4 h (including ~15 min for host DNA depletion, ~75 min for DNA extraction, and ~2.5 h for library preparation and purification of a batch of 12 samples), the approximate range of end-to-end turnaround time from blood culture positivity to report pathogen identification and resistance prediction using BacT-Seq could be estimated at 5.3–19.2 h (median 8.3 h) for the predefined 50X approach, and 4.7–8.4 h (median 6.0 h) for the adaptive approach.

## 4. Discussion

This feasibility study presents BacT-Seq, a novel real-time sequencing platform workflow designed to achieve accurate and fast pathogen identification and antimicrobial susceptibility prediction from positive blood culture in patients with bloodstream infection. We report the results of DNA preparation optimization prior to sequencing, taxonomic identification of pathogens, and several approaches to antimicrobial susceptibility prediction. Like some previously reported feasibility studies on other sequencing pipelines, the results of pathogen identification and antimicrobial susceptibility testing by BacT-Seq were compared to those obtained with conventional culture-based methods (VITEK-MS and VITEK 2 automated systems, respectively) [21,25].

The step of human DNA depletion from the positive blood culture sample using the MITUBE prototype device allowed a clear enrichment in microbial DNA in the extracted DNA preparation, as evidenced by taxonomic characterization of the sequenced DNA by BacT-Seq. Our results agree with those of recent feasibility studies, which implemented MolYsis kits (Molzym, Germany) for host DNA depletion prior to sequencing [21,22,25].

Pathogen identification by BacT-Seq was very fast, achieved in less than 10 min for all but one mono-microbial sample (187/188 [99.5%]). A closer investigation of the one *Campylobacter* sample identified in 47 min of sequencing revealed a very low sequencing throughput, in fact, the lowest sequencing throughput observed in the whole sample set (0.03 Gbp). Similarly, all but one species in poly-microbial samples (24/25 [96.0%]) were identified in less than 15 min by BacT-Seq. Identification of *Klebsiella oxytoca* after 32 min of sequencing was however not related to a low sequencing throughput. 

The concordance of BacT-Seq pathogen identification results with those of the reference method (VITEK-MS) was excellent for mono-microbial samples, reaching 94.7% accuracy (i.e., perfect match) and 96.3% sensitivity (i.e., correct species identification). BacT-Seq was able to detect an additional species not detected by the reference method in four samples and to provide a more accurate identification in ten others, indicating its advantage compared to the reference method. Of the six discrepancies between BacT-Seq and VITEK-MS, four were not true discrepancies as they corresponded to less accurate identifications (species group rank or genus rank), and two were misidentifications at the species level (but correct genus rank) by BacT-Seq. Of the latter, one was a true misidentification (*Serratia ureilytica* by BacT-Seq vs. *Serratia marcescens* by VITEK-MS), while the other (*Aggregatibacter aphrophilus* by BacT-Seq vs. *Aggregatibacter segnis* by VITEK-MS) was not; a closer evaluation showed that *Aggregatibacter segnis* was not present in the sequencing database, leading the BacT-Seq pipeline to assign the sample to the closest species present in the database (i.e., *Aggregatibacter aphrophilus*). Similarly, a closer examination of the sequencing database revealed only one reference genome for *Serratia marcescens*, possibly explaining its misidentification as *Serratia ureilytica* by BacT-Seq. A curation of the BacT-Seq database should solve these issues.

The identification of poly-microbial samples is known to be challenging, regardless of the method of identification used [19,20,21,25,42]. Among the reasons for poly-microbial identification difficulty are the presence of slow-growing pathogens and blood culture conditions, which may favor one species over another [42]. In our feasibility study, BacT-Seq performed reasonably well in identifying the 12 reference polydetections, with 5/12 perfect matches, 5/12 samples for which BacT-Seq missed one species identified by VITEK-MS (the other species being correctly identified), 1/12 sample for which BacT-Seq identified a species missed by VITEK-MS (*Gemella morbillorum*), and 1/12 true discrepancy (*Citrobacter freundii* by BacT-Seq vs. *Citrobacter braakii* by VITEK-MS) together with one species missed by BacT-Seq. *C. freundii* and *C. braaki* are closely related species and often referred to as *C. freundii* complex organisms [43]. Therefore, the *C. freundii/braakii* discrepancy further highlights the need to improve our knowledge database. A closer evaluation of the six BacT-Seq results that missed one species showed the presence of too few sequenced bases for the missed species to allow an accurate identification for four of these six samples. Regarding the other two samples, the presence of the species missed by BacT-Seq was confirmed by an independent method in one sample but not in the second sample. Among these six BacT-Seq results, two missed *P. aeruginosa*, known to be slow growing [44,45], possibly explaining its detection difficulty. Conversely, the *Gemella morbillorum* species identified by BacT-Seq but missed by VITEK-MS is also known to be difficult to identify in cultures [46,47,48,49]. Our study is in line with previous WGS studies also reporting species being missed by sequencing compared to a VITEK-MS reference method [21,25]. Our feasibility study differs however from those studies in that we also observed samples for which the sequencing pipeline identified species missed by the reference method. This might be due to the higher number of clinical samples evaluated in our study (n = 200) compared to theirs (n = 40 and 52, respectively); this could also be due to a difference in performance in pathogen identification between the different pipelines, including differences in the databases used for identification analysis. Further evaluations will be necessary to address this point.

The direct association ARD approach, which aimed at detecting resistance markers within sequenced microbial DNA, showed a moderate specificity (detection of susceptibility phenotypes) together with a poor predictive value for resistance phenotypes (sensitivity ≤ 51.2%). The low sensitivity of this approach indicates that many of the resistance markers that are present in public databases have very low predictive power or are simply missing and that manual curation of these databases is required to improve the performance of the ARD approach. On the other hand, we cannot exclude the possibility that part of the apparent low sensitivity reflects the limitation of the reference AST method to reveal heteroresistance, as discussed below. A future validation study could compare BacT-Seq results to those of population-level phenotyping to determine which genomic signals correspond to minority subpopulations of clinical relevance.

The machine-learning-based ASP approach, which is designed to resistance phenotypes using models trained on genomic and resistance phenotype data, was revealed to be more performant than the ARD approach, at least for some evaluated species such as *E. coli* (96.5% sensitivity, 83.1% specificity). The drawback of our ASP approach as described in the present feasibility study is that it is currently limited to a few common species (*E. coli*, *S. aureus*, *K. pneumoniae*, and *P. aeruginosa*); it must therefore be further developed to cover all relevant species. While the model trained for *E. coli* had a good predictive value, those trained for *S. aureus*, *K. pneumoniae* and *P. aeruginosa* were inefficient in predicting resistance phenotypes (with sensitivities between 44.4% and 50.0%), suggesting that the dataset collected to train these machine learning models was not representative of the full genotype diversity. This is particularly evident for *P. aeruginosa*, which had a high number of wrong R predictions (24/48 [50.0%] R phenotypes predicted as S). The 24 R phenotypes predicted as S by BacT-Seq covered all evaluated antibiotics, indicating an overall poor performance of ASP for *P. aeruginosa*. This poor performance was already noted during cross-validation. Our results are also in line with those of Harris et al., demonstrating a poor performance of R prediction for *P. aeruginosa* using a machine-learning algorithm [25]. This difficulty in predicting resistance from genomic data for *P. aeruginosa* is likely due to the well described complexity by which *P. aeruginosa* regulates the expression of resistance mechanisms, making it challenging to link phenotypes to genotypic changes [45,50,51,52]. As well as this remarkable complexity in resistance mechanisms, the fact that *P. aeruginosa* is slow-growing might add to the difficulty in generating high-quality sequence data [22,44,45]. 

The ASP model for *S. aureus* classified five of the nine reference R phenotypes as S. Of these five misclassifications, three involved the drug erythromycin. Further investigation revealed that the three strains harbored the same resistance marker, which is not covered by the current version of the ASP signature. This highlights the need to improve existing ASP models by including a more diverse and representative range of samples in the training dataset. Finally, although the ASP model for *E. coli* showed a reasonably good specificity (363/437 [83.1%]), 74 S phenotypes were classified as R by BacT-Seq, most of which (62/74 [83.8%]) involved four antibiotics (amoxicillin/clavulanic acid, cefotaxime, ceftazidime, and cefuroxime). A closer look at the probabilities outputted by the model showed that by adjusting the decision threshold, one could highly reduce the number of errors for these drugs.

Altogether, the machine-learning ASP approach for predicting resistance phenotypes from sequencing data is promising. Nevertheless, existing ASP models need to be improved, and new models must be trained to cover additional species before being applicable for clinical use.

It is worth mentioning here that apparent discrepancies between WGS-based and phenotypic AST results may also reflect differences in definitional thresholds—algorithmic vs. biological—rather than analytical inaccuracy. Indeed, interpretation of antimicrobial resistance differs substantially when comparing WGS-based prediction with conventional phenotypic AST. WGS can detect resistance determinants present in low-frequency subpopulations within a heterogeneous bacterial population (i.e., heteroresistance). Under antibiotic pressure, these rare genotypes may rapidly expand, leading to clinical failure despite an initial susceptibility result by conventional AST, illustrating a gradient of resistance. Consequently, what may appear as a “false-positive” resistance call by WGS often represents effective detection of antimicrobial resistance gene-bearing subpopulations that monoclonal phenotypic assays cannot reveal. It should be noted that, besides WGS, other approaches considering the underlying bacterial population structure can capture this resistance gradient. These include genotypic methods such as Droplet Digital PCR (ddPCR) or Deep Amplicon Sequencing and phenotypic methods such as Population Analysis Profiling (PAP) or the recently described Microfluidic Single-Cell AST [53,54].

The median time to result of the ASP approach for the four evaluated species (*E. coli*, *S. aureus*, *K. pneumoniae*, and *P. aeruginosa*) using an optimized adaptive procedure was below 2 h of sequencing, ranging from 0.5 to 4.2 h. Considering that pre-sequencing steps require about 4 h, this represents an approximate turnaround time of 4.7–8.4 h from blood culture positivity to reporting pathogen identification and resistance prediction using BacT-Seq. In comparison, the turnaround time from flagging of a positive blood culture to report generation in the Harris et al. study was 9–17 h [25]. In both cases, time to result is therefore expected to be faster than with conventional AST methods, which usually requires 2–3 days or more [7,8,9].

The main strength of this study is the use of a large number of clinical samples (n = 200) to assess the feasibility of the BacT-Seq platform, notably in comparison to existing feasibility studies [21,25]. Another strength is the comparison of the BacT-Seq results to those of reference methods for identification and susceptibility testing, similarly to other reported feasibility studies [21,25].

Our study presents a few limitations. Although a large number of positive blood culture samples were included in our analysis, samples were selected to cover both most prevalent and rare microbial species, while limiting over-represented ones, resulting in the inclusion of 98.6% bacterial isolates. Future studies should solve this sampling bias by including a large number of unselected samples covering a broader range of pathogens and resistance phenotypes, to validate the applicability and clinical utility of BacT-Seq in a real-world setting. As indicated above, the promising machine-learning-based ASP approach of resistance prediction was limited to four species (*E. coli*, *S. aureus*, *K. pneumoniae*, and *P. aeruginosa*). These four bacterial species represented about half of the isolates included in our cohort; the other half was not evaluated by the ASP approach of the BacT-Seq prototype. ASP signatures covering additional microbial species are currently under development to fill this gap, to improve and broaden BacT-Seq prediction models and increase their applicability in clinical practice. Validation studies of the improved and upgraded BacT-Seq pipeline are under preparation. 

## 5. Conclusions

The BacT-Seq platform allows the fast and accurate identification of pathogens from positive blood cultures. BacT-Seq demonstrated added value in the identification of mono-microbial samples compared to the conventional VITEK-MS approach, while identification of poly-microbial samples remains challenging. Because conventional reference AST is clonal, evaluation of sequencing-based prediction should incorporate population-level validation to fully capture the heteroresistance dynamics enabled by sequencing-based methods. Altogether, machine-learning-based antimicrobial susceptibility prediction is promising but needs further improvement and validation before it can be implemented in clinical routine.

## Figures and Tables

**Table 1:** Description of the 12 samples identified as poly-microbial by the reference method.

Sample Number	Species 1	Species 2	Species 3
1	*Staphylococcus epidermidis*	*Staphylococcus hominis*	
2	*Micrococcus luteus*	*Moraxella osloensis*	
3	*Enterococcus faecium*	*Staphylococcus haemolyticus*	
4	*Bacteroides fragilis*	*Pseudomonas aeruginosa*	*Streptococcus intermedius*
5	*Candida glabrata*	*Candida krusei*	
6	*Enterococcus faecalis*	*Escherichia coli*	
7	*Staphylococcus epidermidis*	*Stenotrophomonas maltophilia*	
8	*Bacteroides fragilis*	*Streptococcus constellatus*	
9	*Citrobacter braakii*	*Enterococcus casseliflavus*	*Klebsiella oxytoca*
10	*Enterococcus faecalis*	*Escherichia coli*	
11	*Pseudomonas aeruginosa*	*Serratia marcescens*	
12	*Enterococcus faecalis*	*Escherichia coli*	

**Table 2:** Description of the four samples identified as mono-microbial by the reference method and as poly-microbial by the BacT-Seq pipeline.

Reference ID	BacT-Seq ID
*Proteus vulgaris*	*Proteus vulgaris* + *Citrobacter koseri*
*Staphylococcus capitis*	*Staphylococcus capitis* + *Staphylococcus haemolyticus*
*Hafnia alvei*	*Hafnia alvei* + *Staphylococcus saprophyticus*
*Streptococcus anginosus*	*Streptococcus anginosus* group + *Streptococcus milleri*

ID, identification.

**Table 3:** Description of mono-microbial samples with more accurate identification with the BacT-Seq pipeline than with the reference method.

Reference ID	BacT-Seq ID	N
*Enterobacter cloacae*	*Enterobacter hormaechei*	6
*Klebsiella pneumoniae*	*Klebsiella quasipneumoniae*	3
*Streptococcus mitis/oralis*	*Streptococcus oralis*	1

ID, identification; N, number of samples per category.

**Table 4:** Description of discrepancies in mono-microbial identifications by the reference method and the BacT-Seq pipeline.

Reference ID	BacT-Seq ID	N
*Streptococcus anginosus*	*Streptococcus anginosus* group	1
*Streptococcus mitis/oralis*	*Streptococcus*	1
*Bacillus circulans*	*Bacillus*	1
*Prevotella buccae*	*Prevotella*	1
*Aggregatibacter segnis*	*Aggregatibacter aphrophilus*	1
*Serratia marcescens*	*Serratia ureilytica*	1

ID, identification; N, number of samples per category.

**Table 5:** Description of the 12 samples identified as poly-microbial by the reference method and comparison with the identification obtained with the BacT-Seq pipeline.

Sample Number	Reference ID	BacT-Seq ID
1	*Staphylococcus epidermidis* *Staphylococcus hominis*	*Staphylococcus epidermidis* *Staphylococcus hominis*
2	*Staphylococcus epidermidis* *Stenotrophomonas maltophilia*	*Staphylococcus epidermidis* *Stenotrophomonas maltophilia*
3	*Enterococcus faecalis* *Escherichia coli*	*Enterococcus faecalis* *Escherichia coli*
4	*Enterococcus faecalis* *Escherichia coli*	*Enterococcus faecalis* *Escherichia coli*
5	*Enterococcus faecalis* *Escherichia coli*	*Enterococcus faecalis* *Escherichia coli*
6	*Bacteroides fragilis* *Streptococcus intermedius* ***Pseudomonas aeruginosa***	*Bacteroides fragilis* *Streptococcus intermedius*
7	*Klebsiella oxytoca* ***Citrobacter braakii*** ***Enterococcus casseliflavus***	*Klebsiella oxytoca* ***Citrobacter freundii***
8	*Bacteroides fragilis* *Streptococcus constellatus*	*Bacteroides fragilis* *Streptococcus constellatus* ***Gemella morbillorum***
9	*Moraxella osloensis* ***Micrococcus luteus***	*Moraxella osloensis*
10	*Enterococcus faecium* ***Staphylococcus haemolyticus***	*Enterococcus faecium*
11	*Candida glabrata* ***Candida krusei***	*Candida glabrata*
12	*Serratia marcescens* ***Pseudomonas aeruginosa***	*Serratia marcescens*

Discordant pathogens are highlighted in **bold**. ID, identification.

**Table 6:** Performance of the ARD resistance prediction by the BacT-Seq pipeline relative to the reference AST method.

ARD Analysis	Sensitivity ^1^ n/N (%)	Specificity ^2^ n/N (%)
Drug-level associations	351/880 (39.9%)	2199/2466 (89.2%)
Family-level associations	451/880 (51.2%)	1931/2466 (78.3%)

^1^ Sensitivity is defined as the proportion of reference R phenotypes correctly predicted as R by ARD (BacT-Seq); ^2^ Specificity is defined as the proportion of reference S phenotypes correctly predicted as S by ARD (BacT-Seq).

**Table 7:** Performance of the ASP resistance prediction models of the BacT-Seq pipeline relative to the reference AST method.

Strain-Specific ASP Model	Sensitivity ^1^ n/N (%)	Specificity ^2^ n/N (%)
*S. aureus*	4/9 (44.4%)	104/105 (99.0%)
*K. pneumoniae*	4/8 (50.0%)	135/143 (94.4%)
*E. coli*	55/57 (96.5%)	363/437 (83.1%)
*P. aeruginosa*	24/48 (50.0%)	97/100 (97.0%)

^1^ Sensitivity is defined as the proportion of reference R phenotypes correctly predicted as R by each of the four strain-specific ASP model (BacT-Seq). ^2^ Specificity is defined as the proportion of reference S phenotypes correctly predicted as S by each of the four strain-specific ASP model (BacT-Seq).

## Data Availability

The original contributions presented in this study are included in the article and Supplementary Material. Further inquiries can be directed to the corresponding author.

## Supplementary Materials

The following supporting information can be downloaded at https://www.mdpi.com/article/10.3390/diagnostics16010133/s1, Table S1: Training dataset used to build the machine-learning-based ASP models.
