# Supplementary material for: BacT-Seq, a Nanopore-Based Whole-Genome Sequencing Workflow Prototype for Rapid and Accurate Pathogen Identification and Resistance Prediction from Positive Blood Cultures: A Feasibility Study
Source: Diagnostics (Basel). 2026 Jan 1;16(1):133. doi: 10.3390/diagnostics16010133 (PMC12785933; doi:10.3390/diagnostics16010133)
Supplement: Supplementary file 1 [file diagnostics-16-00133-s001.zip › diagnostics-3926222-supplementary.pdf]

Table S1. Training dataset used to build the machine-learning-based ASP models.

Species	Antibiotics	nR	nS	n total
S. aureus	erythromycin	656	1314	1970
S. aureus	gentamicin	160	1965	2125
S. aureus	levofloxacin	48	180	228
S. aureus	methicillin	710	1374	2084
S. aureus	tetracycline	210	1712	1922
S. aureus	clindamycin	402	527	929
S. aureus	vancomycin	14	1792	1806
K. pneumoniae	ciprofloxacin	2507	233	2740
K. pneumoniae	ceftazidime	2329	321	2650
K. pneumoniae	amikacin	462	1749	2211
K. pneumoniae	imipenem	1118	1397	2515
K. pneumoniae	meropenem	1157	1191	2348
K. pneumoniae	cefepime	1452	568	2020
K. pneumoniae	aztreonam	1956	253	2209
K. pneumoniae	cefoxitin	1276	741	2017
E. coli	ciprofloxacin	833	1537	2370
E. coli	ceftazidime	490	1647	2137
E. coli	amoxicillin + clavulanic acid	618	1121	1739
E. coli	cefotaxime	296	1235	1531
E. coli	ampicillin	1089	317	1406
E. coli	cefuroxime	299	1174	1473
E. coli	meropenem	75	810	885
E. coli	ceftriaxone	634	373	1007
E. coli	tobramycin	156	624	780
E. coli	levofloxacin	442	101	543
E. coli	chloramphenicol	118	236	354
P. aeruginosa	meropenem	1111	909	2020
P. aeruginosa	ceftazidime	872	1055	1927
P. aeruginosa	levofloxacin	928	701	1629
P. aeruginosa	amikacin	303	1152	1455
P. aeruginosa	tobramycin	640	1065	1705
P. aeruginosa	imipenem	982	630	1612
P. aeruginosa	cefepime	507	766	1273
P. aeruginosa	piperacillin + tazobactam	624	611	1235
P. aeruginosa	ciprofloxacin	811	666	1477
P. aeruginosa	aztreonam	469	467	936
P. aeruginosa	ticarcillin + clavulanic acid	517	164	681

nR, number of genomes associated with an antimicrobial resistance (R) phenotype; nS, number of genomes associated with an antimicrobial susceptibility (S) phenotype; n total, total number of genomes used to train the ASP classifiers.
